# Monocyte platelet aggregates in children with Kawasaki disease- a preliminary study from a tertiary care centre in North-West India

**DOI:** 10.1186/s12969-021-00515-3

**Published:** 2021-03-12

**Authors:** Pandiarajan Vignesh, Amit Rawat, Jitendra Kumar Shandilya, Man Updesh Singh Sachdeva, Jasmina Ahluwalia, Surjit Singh

**Affiliations:** 1 Allergy Immunology Unit, Department of Pediatrics, Advanced Pediatrics Centre, Postgraduate Institute of Medical Education and Research, 160012 Chandigarh, India; 2 Department of Hematology, Postgraduate Institute of Medical Education and Research, 160012 Chandigarh, India

**Keywords:** Kawasaki Disease, Platelet activation, Monocyte platelet aggregates, Flow cytometry, India

## Abstract

**Background:**

Platelet activation is an integral part of pathogenesis of Kawasaki disease (KD). However, there is paucity of literature on flow-cytometry based assessment of platelet activation in KD. We aimed to analyse monocyte-platelet aggregates (MPAs), one of the sensitive markers for platelet activation, by flow cytometry in children with KD.

**Findings:**

In this single-centre prospective study, we have enrolled 14 children with KD and results were compared with age-matched febrile (*n* = 15) and healthy (*n* = 13) controls. After gating monocytes in side-scatter plot, MPAs were identified based on CD14 and CD41 expression. Two (2) ml of blood samples for children with KD were collected at 3 phases of illness- acute stage before start of intravenous immunoglobulin or aspirin, 24 h after completion of IVIg infusion, and 3 months after acute episode of KD.

Children with KD had a significantly higher MPA% values [Median (IQR)- 41.3% (26.6, 52.7)] when compared with febrile [Median (IQR)- 5.98% (2.98-9.72)] and normal [Median (IQR)- 4.48% (2.57-5.59)] controls, *p*<0.01. On follow-up, the MPA% showed a gradual decline in children with KD, but even at 3 months, the value [Median (IQR)- 7.55% (4.15-14.6)] was higher compared to healthy controls [Median (IQR)- 4.48% (2.57-5.59)].

**Conclusions:**

Our results suggest that MPA% was significantly elevated in acute stages in children with KD and activated platelets may continue to persist even after systemic inflammation has subsided. Future studies are warranted whether objective evidence of platelet activation may guide the use of immunomodulatory and anti-platelet therapy in KD.

## Introduction

Kawasaki disease (KD) is a multi-systemic vasculitis that predominantly affects young children [1]. It is considered to be the most frequent cause of acquired heart disease in children in developed countries. Hospital-based data from our centre have also shown a sustained increase in number of cases of KD since the mid-1990 s [2]. Although over 50 years have passed since Dr Tomisaku Kawasaki first reported a case series of this disease, its etiology remains unknown [1]. Approximately 15–25 % of patients with KD may develop coronary artery abnormalities (CAAs) including dilatations and aneurysms if the diagnosis is missed or delayed and treatment not initiated in time. Prompt therapy during the acute phase of disease with intravenous immunoglobulin (IVIg) results in a marked decrease in incidence of CAAs [1].

Vasculitis of systemic small- and medium-sized arteries, especially the coronary arteries, is the main pathological finding in KD and this can result in endothelial cell damage and platelet activation. Activated platelets can in turn secrete several chemokines (platelet factor 4, CXCL5, IL-8, CXCL-7, CCL5, CCL7) and enhance inflammation in vascular walls [3, 4]. Platelet activation is, therefore, considered to play an essential role in pathogenesis of KD.

Markers of platelet activation such as leukocyte-platelet aggregates and P-selectin expression are elevated in coronary artery disease (CAD) and are implicated in progression of cardiovascular events [5]. Among all leukocytes, monocytes have the highest affinity towards platelets to form aggregates [6]. Monocyte-platelet aggregates (MPAs) are even considered a better marker for activated platelets than P-selectin expression [6]. Occurrence of coronary thrombosis and premature arteriosclerosis in KD suggests that excess activation of platelets and coronary vasculitis may play an important role in progression of vascular damage in KD. Though platelet activation has been studied in KD, there is paucity of literature on flow cytometry-based assessment of leukocyte-platelet aggregates in KD and there are no data from developing countries [7–13].

## Findings

### Materials and Methods

Children with KD diagnosed in the Allergy Immunology Unit, Advanced Pediatrics Centre, Postgraduate Institute of Medical Education and Research, Chandigarh, India, from July 2015 to December 2016 were enrolled. Written informed consent was obtained from parents. Diagnosis of KD was based on the American Heart Association (AHA) criteria [1]. Age and sex-matched febrile children who had a diagnosis other than KD were taken as febrile controls. Siblings nearest in age to index patient were taken as healthy controls. In cases where a sibling control was not available or accessible, an age-matched control was enrolled from outpatient clinics.

The Institute Thesis Committee and the Institute Ethics Committee approved the study protocol. The Departmental Review Board has approved the manuscript. Standard treatment protocols were followed for management of KD [1].

### Sample collection

Venepuncture was carried out under aseptic conditions and with minimal occlusion. Two (2) ml of peripheral venous blood was withdrawn into a vacutainer containing 0.106 mol/L of trisodium citrate (blood-citrate ratio 9:1) after discarding initial 1 ml of sample. Samples were collected at three different phases in children with KD- acute stage before administration of IVIg and aspirin; 24 h after administration of IVIg; and approximately 3 months after onset of illness. Febrile and healthy controls had their blood drawn only once in an identical manner.

### Measurement of monocyte‐platelet aggregates

**Fig. 1:**
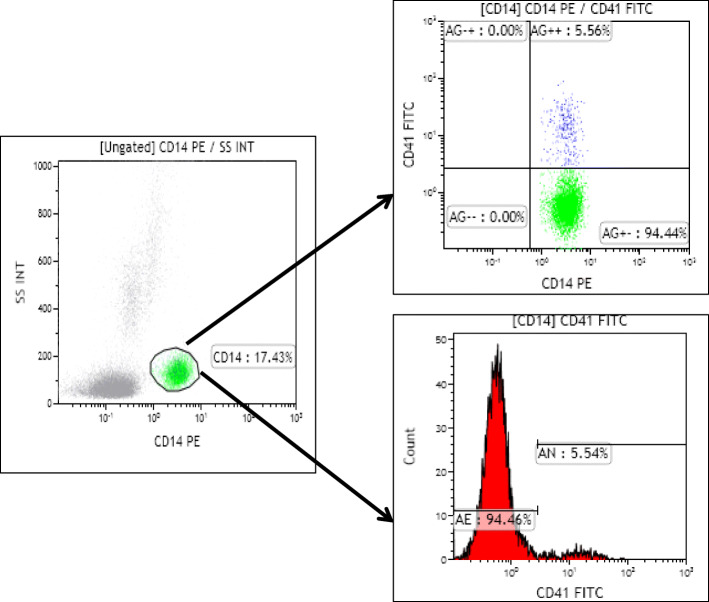
Representative flow cytometry plots that demonstrate gating of monocytes from the side scatter plot of lymphocytes, and analysis of MPA% by identifying monocyte populations that express both CD14 and CD41

Blood samples were processed as quickly as possible (within 2 min) to avoid time-dependent spontaneous aggregation and activation of platelets. Vortexing, centrifugation, and stirring were avoided before fixation of platelets. Fixation of cells and erythrocyte lysis was carried out at room temperature using *Becton Dickinson* FACS® lysing solution. After 10 min of incubation, samples were washed with phosphate-buffered saline and immuno-labelled with appropriate quantities of fluorescein isothiocyanate (FITC) conjugated GP IIb (CD41, BD Pharmingen™, *Catalogue no.*555,466) and PerCP-conjugated CD14 (BD Pharmingen™, *Catalogue no.*555,398) antibodies. After incubation for 20 min at room temperature, cells were acquired on a flow cytometer (Beckman Coulter, Navios). Using *Kaluza*® software, monocytes were gated from lymphocyte side scatter plot. MPAs were identified from monocyte population that expressed both CD14 and CD41 [Fig. 1].

Assessment of MPAs is subject to in vitro platelet activation, and there is a chance for falsely elevated levels of MPAs as a result of this activation. The experiment was carried out initially in 10 healthy controls for standardization of the methodology. Several steps done to prevent in vitro platelet activation include collection of free flow blood without squeezing, immediate fixation of platelets, and minimizing the steps of vortexing and centrifugation during analysis. Efforts were put in to ensure that these precautions were observed during the conduct of this study. Standardization assays were also performed to find out the most appropriate quantity of antibody and the required time for incubation for red cell lysis. During standardization, samples were processed with increasing quantities of antibodies (i.e. 5 µL, 8 µL, 10 µL, 15 µL, and 20 µL) and 8 µL of antibody was found to yield appropriate results.

### Statistical analysis

Means were compared using unpaired student ‘t’ test and analysis of variance (ANOVA). Medians were compared using Mann-Whitney U test and Kruskal Wallis test. Categorical variables were compared by Fisher exact test. A 2-tailed p-value less than 0.05 was considered significant. Statistical analysis was performed with SPSS statistical software version 20.0 (SPSS Inc., Chicago, IL, USA).

## Results

Fourteen [14] children with KD were enrolled during the study period. Fifteen [2] children were taken as febrile controls and 13 as healthy controls. Presumed aetiology of fever in febrile controls was viral upper respiratory tract infections [5], enteric fever [5], staphylococcal abscesses [3], and pneumonia [2]. Median age (interquartile range (IQR)) in cases, febrile controls, and normal controls was 6 (3.0-7.25), 5 (3.6-9.0), and 5.5 (4.15–6.5) years, respectively. Male to female ratio among the 3 groups was 12:2, 13:2, and 11:2, respectively. Median duration of fever in febrile controls was 9 days (range: 5–14 days), which is significantly lower than median duration of fever in children with KD, 13.5 days (range: 7–21 days), *p* = 0.024. (Table 1). Two children had transient coronary artery abnormalities (CAAs) in form of brightness of coronary arteries and coronary ectasia. One child (7-month-old boy) developed giant coronary aneurysms in left main, left anterior descending, and right coronary arteries. All patients received IVIg (2 g/kg) infusion for treatment for KD. Two patients also received IV infliximab infusion (5 mg/kg)– one child with giant coronary artery aneurysm and the other with severe systemic disease and transient CAA. Antiplatelet dose of aspirin was continued for 3 months in one child as he persisted in having elevated platelet counts (640 × 10^9^/L).

**Table 1:** Baseline clinical and laboratory characteristics of children with Kawasaki disease at enrolment (n = 14)

Clinical parameter	Result
Median age (IQR)	6 years (3.0, 7.25)
Male: Female	12: 2
Duration of fever [median (IQR)]	13.5 days (8.0, 15.0)
No. of cases with incomplete KD (%)	5 (35.7 %)
Hemoglobin in g/dL [median (IQR)]	9.2 (7.6, 11.3)
White cell counts (x 10^9^/L) [median (IQR)]	14.15 (10.6, 21.2)
Absolute neutrophil counts (x 10^9^/L) [median (IQR)]	7.9 (6.2, 12.4)
Platelet counts (x 10^9^/L) [median (IQR)]	385.5 (233.5, 598.5)
Erythrocyte sedimentation rate at 1st hour (mm) [median (IQR)]	45 (28.5, 69.5)
C-reactive protein in mg/L [median (IQR)]	48.5 (23, 81.8)

*IQR* Inter-quartile range; *KD *Kawasaki disease

**Fig. 2:**
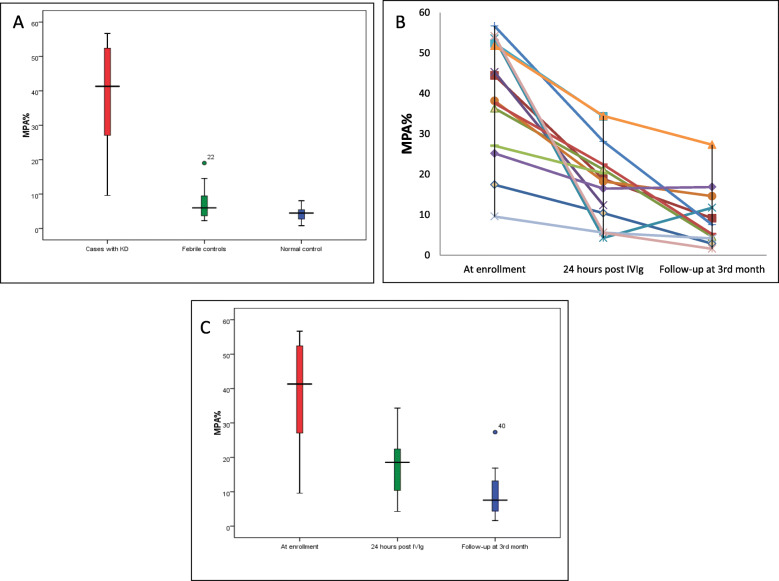
**a**. Boxplot showing MPA% between children with KD at enrolment (*n* = 14) [Median (IQR)- 41.3 (26.6–52.7)], febrile controls (*n* = 15) [Median (IQR)- 5.98 (2.98–9.72)], and normal controls (*n* = 13) [Median (IQR)- 4.48 (2.57–5.59)], *p* < 0.001; **b**. Trend of serial values MPA% among all 14 children with KD; **c**. Boxplot showing MPA% between cases at enrolment (*n* = 14) [Median (IQR)- 41.31 (26.6-52.69)], 24 h post-IVIg (*n* = 14) [Median (IQR)- 18.55 (9.2-22.99)], and at 3rd month follow-up (*n* = 11) [Median (IQR)- 7.55 (4.15–14.6)], *p* < 0.001;

MPA% values were significantly high in cases [Median (IQR)- 41.3 % (26.6–52.7)] when compared to febrile [Median (IQR)- 5.98 % (2.98–9.72)] and normal controls [Median (IQR)- 4.48 % (2.57–5.59)], *p* < 0.01 (Fig. 2 a). Median (IQR) absolute monocyte counts of children with KD and febrile controls were 1327.5/cu.mm (937.5, 1515.25) and 1184/cu.mm (880, 2160), respectively (*p* = 0.83). Median (IQR) MPA counts of patients with KD was 530.74/cu.mm (408.87, 614.37) compared to 96.39/cu.mm (25.08, 121.57) in febrile controls, which was statistically significant (*p* < 0.001). A significant drop in MPA% was noted after IVIg therapy. Serial MPA% in patients with KD showed a consistent drop in values from the time of diagnosis until the 3rd month of follow-up (Fig. 2b c).

No change in trend of fall of MPA% was noted even after exclusion of patients who were continued on aspirin (Fig. 2d). MPA% values at 3 months post-diagnosis of KD [Median (IQR)- 7.55 % (4.15–14.6)] were higher compared to normal controls [Median (IQR)- 4.48 % (2.57–5.59)], *p*-0.072.

No significant differences in MPA% were noted between patients with and without CAAs (Table 2). There was also no significant difference in MPA% values between patients with complete and incomplete presentation of KD (Table 3). Median (IQR) MPA% in children with KD with fever duration ≤ 10 days (*n* = 5) was 38.18 % (31.7, 49.5) compared to 44.4 % (31.7, 53) in children with fever duration > 10 days (*n* = 9), which is not statistically significant (*p* = 0.79).

**Table 2:** Median percentage values of MPA (CD14 + CD41+) between children with KD patients with and without coronary artery abnormalities (CAA)

Category	Children with CAA (*n* = 3)	Children without CAA (*n* = 11)	*P* value
At enrolment [median (Range)]	38.18 (17.42–56.67)	44.44 (9.58–54.2)	1.000
24 h after IVIg [median (Range)]	18.26 (10.4-28.13)	18.84 (4.27–34.32)	0.885
Follow-up at 3rd month [median (Range)]	7.55 (2.84–14.6)	7.17 (1.59–27.32)	0.921

**Table 3:** Median percentage values of MPA (CD14 + CD41+) between children with complete (*n* = 8) and incomplete KD (*n* = 6)

Category	Children with complete KD (*n* = 9)	Children with incomplete KD (*n* = 5)	*P* value
At enrolment [median (Range)]	45.3 (27.08–56.67)	25.17 (9.58–53.62)	0.083
24 h after IVIg [median (Range)]	20.28 (5.56–34.32)	10.4 (4.27–22.4)	0.112
Follow-up at 3rd month [median (Range)]	8.34 (1.59–27.32)	5.21 (2.84–16.87)	0.792

## Discussion

Platelet function abnormalities in KD were first reported by Yamada et al. in 1978 [14]. Studies in platelet activation markers in KD have subsequently been undertaken in Japan, USA, and Italy (Table 4). Genetic risk factors and clinical presentation of KD differ among various populations and ethnicities. Hence platelet reactivity in KD can also be expected to be different in different populations. The important differences in clinical presentation of KD at Chandigarh, North India observed were the relatively higher rates of KD in children > 5 years of age compared to other population, and the relatively earlier occurrence of periungual peeling and thrombocytosis during acute phase of illness [2]. Present study has been undertaken to analyse one of the most sensitive platelet activation markers, i.e. MPAs, by flow cytometry in a North Indian cohort of KD. To the best of our knowledge, MPAs has not been studied in acute and subacute phases of KD till date, and our study is perhaps the first attempt along these lines.

**Table 4:** Comparison of studies on platelet functions in Kawasaki disease reported in English literature

Study, year, and Country	Platelet activation marker studied	Study population characteristics	Comments
Yokoyama et al. (1980) Japan [8]	Platelet aggregation by optical density method	23 patients with KD (Age range: 3 months-6 years; M:F = 14:9);	Platelet aggregation in patients with untreated KD (82.4 ± 11.6 %) significantly higher than normal controls (69 ± 5 %) and patients with KD treated with low dose aspirin (66.8 ± 9.6 %). Platelet aggregation increased from 2nd week of illness.
Burns et al. (1984) USA [12]	Plasma beta-thromboglobulin	31 patients with KD	Levels significantly higher in patients with coronary aneurysms compared to patients without aneurysms measured 3 weeks after fever (72.3 ng/ml vs. 29.4 ng/ml respectively, *p* < 0.002).
Taki et al. (2003) Japan [7]	Platelet aggregation by particle counting method (mV count)	104 children with KD (mean age: 2.1 ± 1.9 years; M:F = 15:11); 9 normal controls	Spontaneous platelet aggregation was higher in patients with KD (46.6 × 10^3^±13.2 × 10^3^) compared to normal subjects (9.4 × 10^3^±3 × 10^3^). It reduced significantly after IVIg therapy (22.8 × 10^3^±6.6 × 10^3^).
Ueno et al. (2009) Japan [13]	Platelet VEGF by ELISA	80 patients with KD (Mean age: 2.1 ± 1.8 years, M:F = 43:37); 26 controls	Levels significantly high in KD (18.8 ± 10.1 × 10^− 8^ pg) compared to controls (8.1 ± 3.0 × 10^− 8^ pg). The levels decreased in IVIg responders and remained elevated in IVIg non-responders.
Yahata et al. (2014) Japan [10]	Platelet derived microparticles by ELISA	18 patients with acute KD (mean age: 2 years 7 months) in whom 14 received IVIg therapy; 33 children as febrile controls	Levels were significantly high in acute phase of KD (43.9 ± 13.5 U/ml) compared to febrile controls (15.4 ± 6.8 U/ml). The levels significantly came down with IVIg therapy and it rebounded after discontinuation of aspirin in 8 patients.
Laurito et al. (2014) Italy [9]	Monocyte platelet aggregates by flow cytometry	14 patients with past history of KD (mean follow-up: 76 ± 58 months; M:F = 9:5); 14 controls	Mean %MPAs were similar in patients and controls even after ADP stimulation (18.3 ± 1.9 % vs. 17.2 ± 1.5 %; p = 0.09). CD41 expression in MPA gate was higher in KD than controls after ADP stimulation (19.3 ± 1.3 % vs. 17 ± 1.7; *p* < 0.001).
Ueno et al. (2015) Japan [11]	Neutrophil-platelet aggregates by flow cytometry	40 patients with KD (median age: 1.75 years, M:F ≈ 1:1); 7 febrile controls, and 9 normal controls	Percentage of neutrophil-platelet aggregates were significantly high in KD compared to febrile and normal controls. Rate of decrease in aggregates was significantly high in patients who received prednisolone + IVIg compared to patients who had received IVIg alone.
Present study (2019) India	Monocyte platelet aggregates by flow cytometry	14 patients with acute KD (Median age: 6 years; M:F = 6:1); 15 febrile controls and 13 normal controls	Median %MPA significantly higher in KD compared to febrile and normal control (41.3 vs. 5.9 vs. 4.5 %; *p* < 0.001). Levels significantly came down 24 h after IVIg therapy (18.6 %; *p* < 0.001).

*KD* Kawasaki Disease; *MPA* Monocyte-platelet aggregates; *IVIg* Intravenous immunoglobulin; *VEGF* Vascular endothelial growth factor; *ELISA* Enzyme linked immunosorbent assay; *ADP* Adenosine diphosphate

Levels of MPA% were found to be significantly elevated in children with KD compared to febrile controls and healthy controls in our study (median levels of 41.3 %, 5.98 %, and 4.48 % respectively, *p* < 0.001). MPA% levels significantly decreased after treatment with IVIg (median: 41.3 % vs. 18.5 %, *p* < 0.001) [Fig. 2]. Elevations in MPA% suggest excess activation of platelets in KD compared to other common childhood febrile illnesses. Previous studies report that other platelet activation markers (e.g. neutrophil-platelet aggregates, platelet-derived microparticles, platelet VEGF levels, betathromboglobulin levels, PF4 levels, and platelet CD62P expression) are also elevated in acute phase of KD [7–13]. Our study had a high proportion of children with incomplete KD (35.7 %), which is in line with other recent studies from Asia [15, 16]. Median duration of fever in our cohort of children with KD was 13.5 days that is higher compared to the European and North American cohorts [17, 18]. Duration of fever may have impacted MPA% values in children with KD, as most of our patients were diagnosed more than 10 days after the disease onset when the platelet counts begin to increase. However, comparison of MPA% between children with fever duration ≤ 10 days and > 10 days did not show any statistically significant difference. Ueno et al. studied neutrophil-platelet aggregates (NPAs) in Japanese children with KD who had a median duration of fever of 4 days and had shown that NPAs% levels were significantly higher in KD compared to febrile controls [11].

Median levels of MPA% on follow-up at 3 months were lower compared to levels measured 24 h after IVIg therapy, but higher than values obtained in age and sex-matched controls. It suggests that patients with KD may have a prolonged endotheliitis even after control of systemic inflammation with IVIg therapy. Laurito et al. analyzed MPAs in patients with KD several years after the acute phase (mean interval- 76 months). Authors reported that CD41 expression at baseline and after ADP stimulation was significantly higher in patients compared to controls. However, MPA% levels were not significantly different between two populations [9]. In a recent study by Yahata et al., platelet-derived microparticles were found to have rebound elevations in 8 of 14 patients with KD after discontinuation of aspirin [10]. In our study also, we found rebound elevation in MPA% levels in 2 patients at 3 months of follow-up. However, majority of patients had lower levels even after discontinuation of aspirin.

Ueno et al. reported that levels of NPAs were significantly higher in patients with CAAs when compared with patients without CAAs [11]. In our study, levels of MPA% were not different between patients with CAAs (*n* = 3) and those without CAAs (n = 11). Apparent discrepancy in results could be explained by differences in ethnicities of the study populations, small sample size in both studies, and differences in methodology of assessment of platelet activation. Ueno et al. also reported that levels of neutrophil-platelet aggregates were significantly lower in patients with KD treated with both IVIg and oral prednisolone than in patients who received IVIg alone [11]. Corticosteroids were not used in any patients in our cohort.

A limitation of our study is the small sample size. This is understandable considering the fact that the protocol had to be completed in a limited span of time. Being a single centre study, there was uniformity in diagnosis and treatment protocols followed for management. Efforts were put in to standardize laboratory assays for MPAs before commencement of the study. We could use only single surface marker-CD14 for identification of monocytes in our study, as it is expressed both in classical and non-classical monocytes [19]. CD14 can also be weakly expressed in some neutrophils and B cells, so, monocytes alone may not have been accurately captured in our experiment. Use of an imaging flow cytometry could have precisely showed the aggregates of monocytes and platelets [20], however, we could not use this modality in our study. Our patient cohort had a high proportion of children with KD who had fever of more than 10 days duration (64.3 %). More studies are needed in future to decipher the platelet activation status in early stages of KD.

To conclude, MPA% was significantly elevated in our cohort of children with KD when compared with age and sex-matched febrile and healthy controls. Future long-term studies are warranted to find out whether elevated MPAs in KD would have any clinical implications. This may provide a theoretical basis for additional immunosuppressive and/or antiplatelet therapy in KD.

## Data Availability

The datasets used and/or analyzed during the current study are available from the corresponding author on reasonable request.

## Abbreviations

KD: Kawasaki disease
MPA: Monocyte-platelet aggregates
IVIg: Intravenous immunoglobulin
CAD: Coronary artery disease
AHA: American Heart Association

## Declarations

### Authors’ information

The study was presented in the 24th European Pediatric Rheumatology Congress held at Athens, Greece and the abstract was published in the conference proceedings.

Ref: Proceedings of the 24th Paediatric Rheumatology European Society Congress: Part one. Pediatr Rheumatol Online J 2017;15(Suppl 2):64.

## References

[1] Newburger JW, Takahashi M, Gerber MA, Gewitz MH, Tani LY, Burns JC (2004). Diagnosis, treatment, and long-term management of Kawasaki disease: a statement for health professionals from the Committee on Rheumatic Fever, Endocarditis and Kawasaki Disease, Council on Cardiovascular Disease in the Young, American Heart Association. Circulation.

[2] Singh S, Aulakh R, Bhalla AK, Suri D, Manojkumar R, Narula N, Burns JC (2011). Is Kawasaki disease incidence rising in Chandigarh, North India?. Arch Dis Child.

[3] Orenstein JM, Shulman ST, Fox LM, Baker SC, Takahashi M, Bhatti TR (2012). Three linked vasculopathic processes characterize Kawasaki disease: a light and transmission electron microscopic study. PLoS One.

[4] Chatterjee M, Geisler T (2016). Inflammatory Contribution of Platelets Revisited: New Players in the Arena of Inflammation. Semin Thromb Hemost.

[5] Furman MI, Benoit SE, Barnard MR, Valeri CR, Borbone ML, Becker RC (1998). Increased platelet reactivity and circulating monocyte-platelet aggregates in patients with stable coronary artery disease. J Am Coll Cardiol.

[6] Gerrits AJ, Frelinger AL, Michelson AD (2016). Whole Blood Analysis of Leukocyte-Platelet Aggregates. Curr Protoc Cytom.

[7] Taki M, Kobayashi M, Ohi C, Shimizu H, Goto K, Aso K (2003). Spontaneous platelet aggregation in Kawasaki disease using the particle counting method. Pediatr Int.

[8] Yokoyama T, Kato H, Ichinose E (1980). Aspirin treatment and platelet function in Kawasaki disease. Kurume Med J.

[9] Laurito M, Stazi A, Delogu AB, Milo M, Battipaglia I, Scalone G (2014). Endothelial and platelet function in children with previous Kawasaki disease. Angiology.

[10] Yahata T, Suzuki C, Yoshioka A, Hamaoka A, Ikeda K (2014). Platelet activation dynamics evaluated using platelet-derived microparticles in Kawasaki disease. Circ J.

[11] Ueno K, Nomura Y, Morita Y, Eguchi T, Masuda K, Kawano Y (2015). Circulating platelet-neutrophil aggregates play a significant role in Kawasaki disease. Circ J.

[12] Burns JC, Glode MP, Clarke SH, Wiggins J, Hathaway WE (1984). Coagulopathy and platelet activation in Kawasaki syndrome: Identification of patients at high risk for development of coronary artery aneurysms. J Pediatr.

[13] Ueno K, Nomura Y, Hashiguchi T, Masuda K, Morita Y, Hazeki D (2010). Platelet vascular endothelial growth factor is a useful predictor for prognosis in Kawasaki syndrome. Br J Haematol.

[14] Yamada K, Fukumoto T, Shinkai A, Shirahata A, Meguro T (1978). The platelet functions in acute febrile mucocutaneous lymph node syndrome and a trial of prevention for thrombosis by antiplatelet agent. Nihon Ketsueki Gakkai Zasshi.

[15] Xie LP, Yan WL, Huang M, Huang MR, Chen S, Huang GY, et al. Epidemiologic Features of Kawasaki Disease in Shanghai From 2013 Through 2017. J Epidemiol. 2019 Sep 21. doi:10.2188/jea.JE20190065. Epub ahead of print. 10.2188/jea.JE20190065 PMC7492704 31548437

[16] Kim GB, Park S, Eun LY, Han JW, Lee SY, Yoon KL (2017). Epidemiology and Clinical Features of Kawasaki Disease in South Korea, 2012–2014. Pediatr Infect Dis J.

[17] Skochko SM, Jain S, Sun X, Sivilay N, Kanegaye JT, Pancheri J, et al. Kawasaki Disease Outcomes and Response to Therapy in a Multiethnic Community: A 10-Year Experience. *J Pediatr* 2018;203:408 – 15.e3. 10.1016/j.jpeds.2018.07.090 30268398

[18] Tulloh RMR, Mayon-White R, Harnden A, Ramanan AV, Tizard EJ, Shingadia D (2019). Kawasaki disease: a prospective population survey in the UK and Ireland from 2013 to 2015. Arch Dis Child.

[19] Ong SM, Teng K, Newell E, Chen H, Chen J, Loy T (2019). A Novel, Five-Marker Alternative to CD16-CD14 Gating to Identify the Three Human Monocyte Subsets. Front Immunol.

[20] Hui H, Fuller KA, Erber WN, Linden MD (2017). Imaging flow cytometry in the assessment of leukocyte-platelet aggregates. Methods.

